# Dementia and mortality in older adults: A twin study

**DOI:** 10.1002/alz.13553

**Published:** 2023-12-11

**Authors:** Jung Yun Jang, Christopher R. Beam, Ida K. Karlsson, Nancy L. Pedersen, Margaret Gatz

**Affiliations:** ^1^ Institute for Memory Impairments and Neurological Disorders University of California Irvine Irvine California USA; ^2^ Department of Psychology University of Southern California Los Angeles California USA; ^3^ Davis School of Gerontology University of Southern California Los Angeles California USA; ^4^ Department of Medical Epidemiology and Biostatistics Karolinska Institutet Stockholm Sweden; ^5^ Center for Economic and Social Research University of Southern California Los Angeles California USA

**Keywords:** dementia, genetic and environmental factors, mortality, twin studies

## Abstract

**INTRODUCTION:**

Dementia predicts increased mortality. We used case‐control and co‐twin control models to investigate genetic and shared environmental influences on this association.

**METHODS:**

Case‐control design, including 987 twins with dementia and 2938 age‐ and sex‐matched controls in the Swedish Twin Registry. Co‐twin control design, including 90 monozygotic (MZ) and 288 dizygotic (DZ) twin pairs discordant for dementia. To test for genetic and environmental confounding, differences were examined in mortality risk between twins with dementia and their matched or co‐twin controls.

**RESULTS:**

Twins with dementia showed greater mortality risk than age‐ and sex‐matched controls (HR = 2.02 [1.86, 2.18]). Mortality risk is significantly elevated but attenuated substantially in discordant twin pairs, for example, comparing MZ twins with dementia to their co‐twin controls (HR = 1.48 [1.08, 2.04]).

**DISCUSSION:**

Findings suggest that genetic factors partially confound the association between dementia and mortality and provide an alternative hypothesis to increased mortality due to dementia itself.

**Highlights**

- We studied dementia and mortality in twin pairs discordant for dementia.
- People without dementia outlived people with dementia.
- Identical twins with dementia and their co‐twin controls had similar survival time.
- Findings suggest genotype may explain the link between dementia and mortality.

## 1 BACKGROUND

Abundant research data indicate that one consequence of dementia is shortened lifespan.^1^, ^2^ Typically, these studies compare survival from the point of dementia diagnosis to survival among people without dementia from the same or similar study population (e.g.,^3^). Some studies have examined factors associated with increased mortality in dementia, such as comorbidities^4^ and antipsychotic use.^5^ Information from such studies may clarify expectations for caregivers as well as those diagnosed with dementia for end‐of‐life planning. However, the genetic and environmental mechanisms underlying dementia and mortality are likely complex, and the research evidence to date highlights competing questions: Does dementia itself shorten lifespan? Or do genetically influenced characteristics confound their association? The answers to these questions may provide new understandings of disease mechanisms and offer practical guidance to individuals diagnosed with dementia and their families.

The present study investigated survival after dementia onset using a population‐based sample of Swedish twins. No study has examined whether the association between dementia and increased mortality is attributable to genetic background or environmental influences in common to dementia and mortality or whether the association chiefly reflects the ways in which having dementia shortens the lifespan. Genetic variation contributes to variability in lifespan^6^ and dementia risk.^7^, ^8^ Genetically informed research designs, such as monozygotic (MZ) and dizygotic (DZ) twin models, allow for stronger tests of the effect of dementia diagnosis on lifespan because they statistically control for unmeasured genetic and environmental confounds.

We employed a co‐twin control design^9^, ^10^ in the present study to control for genetic and shared environmental influences. The logic of the co‐twin control design is based on the conditions of MZ twins sharing 100% of their genotype and DZ twins on average sharing 50% of their genotype, while all twins share rearing environments. If genetic background underlies the association between dementia and mortality, differences in mortality between twins with dementia and their disease‐free co‐twins are expected to be larger in DZ pairs discordant for dementia compared to MZ twin pairs. Differences in mortality between members of MZ pairs discordant for dementia directly tests the effect of unique environmental exposures (e.g., effects of dementia itself) on mortality, as effects of genotype are completely removed.

**RESEARCH IN CONTEXT**

1. **Systematic review**: The authors identified key studies of dementia and mortality from systematic reviews, meta‐analyses, and a review of existing literature using relevant search terms in databases. The authors evaluated evidence, focusing on the use of large representative samples and robust analytic approaches to formulate the research question and aid in the interpretation of the results.
2. **Interpretation**: Our findings provide novel evidence of genetic influences in the association between dementia and increased mortality that have not been investigated previously. Findings suggest that co‐twins’ mortality risk following first twins’ diagnosis of dementia is in part familial, as excess risk affects both twins with dementia and their disease‐free co‐twins.
3. **Future directions**: Although the current study sample is representative of the Swedish population, findings should be replicated in other samples. Future endeavors may also expand the findings by delineating mechanisms by which specific genes affect both dementia and mortality.

In the current study, we examined the dementia‐mortality associations in (1) twins with dementia and unrelated controls (case‐control analysis similar to prior literature), and (2) DZ and MZ twins with dementia and their cognitively intact co‐twins (co‐twin control analysis).^11^ We hypothesized the following:

1. The dementia‐mortality association would be greatest in the case‐control analysis.
2. The dementia‐mortality association would be attenuated in both the DZ and MZ co‐twin control analyses, and particularly in the MZ co‐twin control analysis, which controls for all genetic and environmental risk factors that make MZ twins alike.
3. Still, the association between dementia diagnosis and mortality in the MZ co‐twin control analysis would be significant, suggesting that consequences of having dementia (e.g., poor health behaviors due to cognitive impairment) would account for increased risk for mortality in twins with dementia.

## 2 METHODS

Data collection procedures were reviewed and approved by the Regional Ethics Board at Karolinska Institutet and by the Institutional Review Board at the University of Southern California. We certify that the current study was conducted in accordance with the ethical standards of the 1964 Declaration of Helsinki and its later amendments.

### 2.1 Participants and design

The sample was drawn from four studies based on the Swedish Twin Registry (STR)^12^, ^13^: Aging in Women and Men (GENDER),^14^ Origins of Variance in the Oldest Old: Octogenarian Twins (OCTO‐Twin),^15^ the Swedish Adoption Twin Study of Aging (SATSA),^16^, ^17^ and the Study of Dementia in Swedish Twins (HARMONY).^18^ Twins were born between 1886 and 1944 and were assessed between 1984 and 2015. The STR includes in principle all twins born in Sweden during the included birth years. Moreover, twins have been shown to be representative of the general population from the respective birth cohorts,^19^, ^20^, ^21^, ^22^ and the prevalence of dementia in the STR and the general population was comparable.^18^ Participants are all ethnically Swedish. The sample includes women and men and is heterogeneous in indicators such as education and social condition. For instance, 64.4% of the sample had only primary education, 22.1% had primary education but did not graduate from high school, and 13.5% graduated from high school or had higher education.

Zygosity for same‐sex twins in the STR was initially based on survey questions about physical similarity as children between twins in a pair. Once blood samples were collected for genotyping, they were also used for zygosity confirmation. The accuracy of zygosity taken from self‐reported similarity questions in the STR has been found to be 95% to 98% against genotyping.^23^ In the present study, 99% of zygosities have been confirmed by DNA.

Dementia status is established through clinical workup with additional follow‐up via linkage to the population‐based registries (see Section 2.2.1, Dementia ascertainment). The current study adopted a stepwise approach to examine the risk for mortality in relation to dementia diagnosis, using unrelated case‐control and co‐twin control designs.

#### 2.1.1 Unrelated case‐control design

**FIGURE 1:**
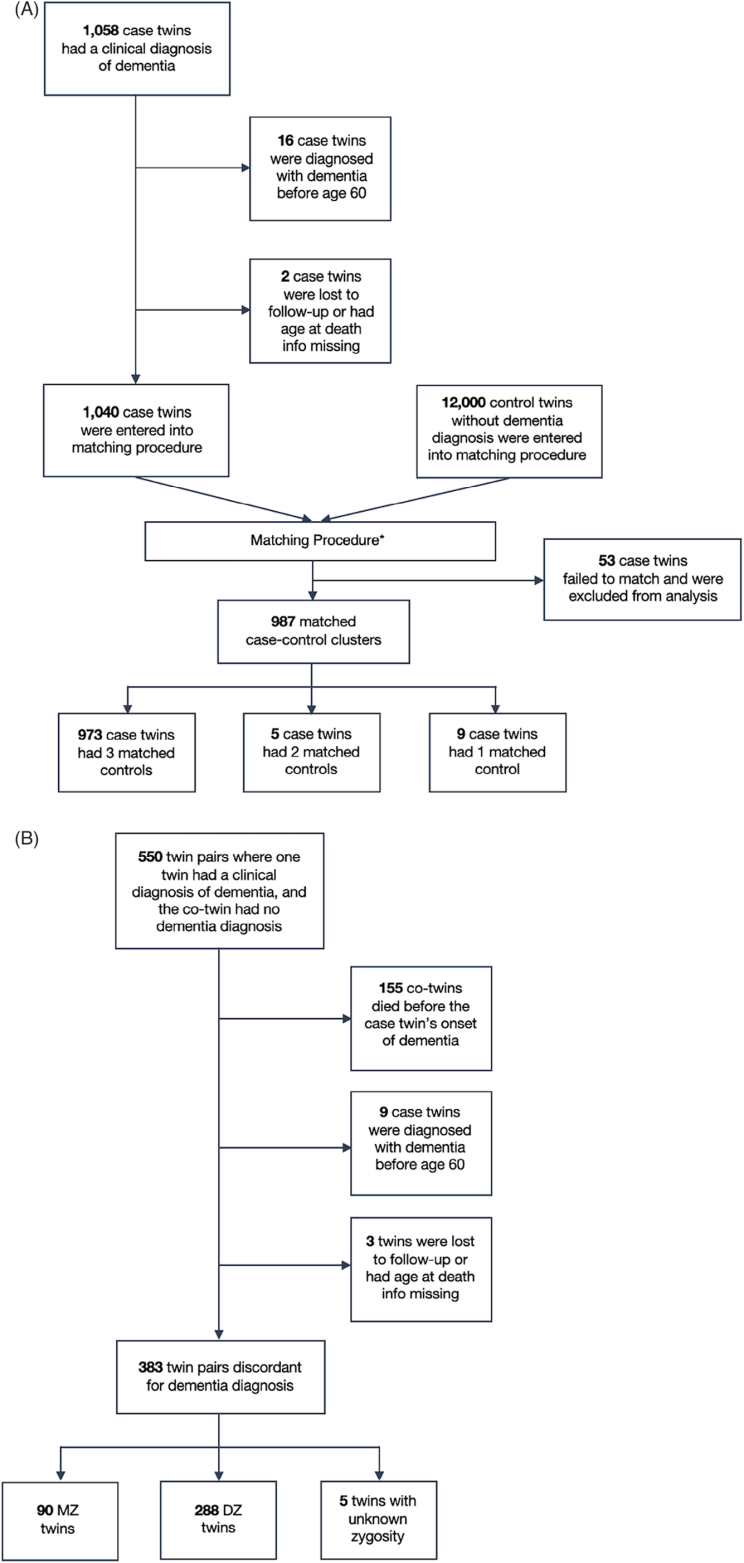
Flow charts describing the inclusion of study samples for case‐control design (A), and co‐twin control design (B). DZ, dizygotic; MZ, monozygotic. ^*^Criteria used for matching in the case‐control design: Controls were (1) born within 2 years of the case twin, (2) of the same sex, (3) not the case twin's co‐twin, and (4) part of the study at the time of the case twin's age at onset (i.e., were not deceased or lost to follow‐up before the dementia onset).

The unrelated case‐control design compared individuals with dementia (“cases”) to unrelated individuals without dementia (“controls”), with a target of three controls for each case. Twins diagnosed with dementia were matched to a unique set of twins without dementia who remained free of dementia from initial evaluation through registry follow‐up. Matching criteria were (1) birth year (± 2 years), (2) sex, (3) not the case twin's co‐twin, and (4) not deceased or lost to follow‐up before the dementia onset of the case. Of the 1058 twins with a clinical diagnosis of dementia, 18 were excluded due to age at onset younger than 60 years (*n* = 16), attrition for an unknown reason (*n* = 1), or missing death date information (*n* = 1). If both members of a twin pair were diagnosed with dementia, both were included as cases. If both members of a twin pair were without dementia diagnosis, both were eligible as controls and included in the matching. Consequently, 1040 cases were entered into the matching process along with all available 12,000 controls from the study sample, which generated a total of 987 case and matched control clusters. Of these, 973 clusters had three matched controls per case, five had two matched controls per case, and nine had one matched control per case. Fifty‐three cases failed to match and were excluded from the analysis (Figure 1A). Demographic characteristics of the case‐control sample are described in Table 1.

**TABLE 1:** Sample characteristics for cases and matched controls in case‐control analysis.

		Cases (*n* = 987)	Controls (*n* = 2938)	*t* or *χ* ^2^	*p*
Birth year	range	1886–1942	1886–1944	–	–
Female	*n* (%)	631 (63.9)	1870 (63.7)	0.03	0.87
Age at dementia onset	mean (SD)	78.22 (7.19)	–	–	–
Education^a^	mean (SD)	1.37 (0.92)	1.73 (1.34)	7.91	<0.001
Self‐rated health^b^	mean (SD)	53.40 (10.12)	53.82 (11.12)	−0.89	0.37
Deceased	*n* (%)	969 (98.2)	2643 (90.0)	67.98	<0.001
Time to death^c^	mean (SD)	7.85 (4.94)	10.21 (6.71)	10.02	<0.001
Time to censoring^d^	mean (SD)	10.18 (6.42)	18.64 (6.50)	5.37	<0.001

^a^ International Standard Classification of Education (ISCED) codes.

^b^ Data are shown in T‐scores (mean = 50, SD = 10).

^c^ In each case and matched controls cluster, time to death was calculated based on age at death − case twin's age at dementia onset, for twins who are deceased.

^d^ In each case and matched controls cluster, time to censoring was calculated based on age at the latest death data update in the Swedish Twin Registry (December 31, 2016) − case twin's age at dementia onset, for twins who are right‐censored.

#### 2.1.2 Co‐twin control design

For co‐twin control analyses, we identified twin pairs discordant for dementia, where one twin received a clinical diagnosis of dementia (“cases”), and the co‐twin had no dementia, either at initial evaluation or through registry follow‐up (“controls”). Of the 550 dementia discordant pairs, 155 were excluded because the co‐twin died before the case's onset of dementia, nine were excluded because the case's age at onset was younger than 60 years, and three were excluded due to attrition for an unknown reason or missing death age information. This resulted in a total of 383 dementia discordant twin pairs, including 288 DZ pairs and 90 MZ pairs (Figure 1B). DZ pairs included same‐sex as well as opposite‐sex pairs. Demographic characteristics of the co‐twin control samples are described in the Supplemental Tables by zygosity.

#### 2.1.3 Post hoc analysis: MZ co‐twin controls versus unrelated MZ controls

We performed a post hoc analysis to compare mortality risk in controls from the MZ co‐twin control analyses and unrelated MZ controls from dementia‐free pairs matched on age and sex. Differences in mortality risk between MZ co‐twin controls and unrelated MZ controls would corroborate evidence for genetic confounding in the association between dementia and mortality. Although MZ co‐twin controls do not have dementia, if they have higher risk for mortality compared with members of MZ twin pairs where neither twin has a diagnosis of dementia, the result would support genetic confounding.

We identified 706 MZ pairs from the study sample for which neither member had a dementia diagnosis. After five individuals were excluded due to attrition for an unknown reason or missing death age data, 1407 MZ twins were entered into the same matching procedure as in the unrelated case‐control design described in Section 2.1.1. This procedure found three unrelated MZ controls for 84 MZ co‐twin controls and two for six MZ co‐twin controls. Thus, the sample consisted of 90 MZ co‐twin controls and 264 unrelated MZ controls for a total of 90 clusters.

### 2.2 Measures

#### 2.2.1 Dementia ascertainment

All twins underwent cognitive screening at each point of contact. HARMONY is a one‐time census of the entire STR aged 65 and older, entailing a two‐stage dementia assessment. The first stage was telephone cognitive screening of participants using the TELE^24^ and informant interviews using the Blessed Dementia Rating Scale.^25^ Following telephone cognitive screening of the entire population, all twins who performed poorly, their co‐twins, and a sample of twins who screened as cognitively intact were invited to in‐person dementia evaluation including physical and neurological examination, medical history based on medical record review and informant interview, a neuropsychological assessment (Mini‐Mental State Examination, word list immediate and delayed recall, verbal fluency, block design, figure copying, judgment, information, digit symbol, prospective memory, confrontation naming, and delayed recall of objects and locations), blood panels, and neuroimaging. A consensus panel reviewed all twins and assigned dementia diagnoses. None of the twins who screened as cognitively intact were deemed to have cognitive impairment; on this basis, diagnoses of normal cognition were assigned to all those whose screening indicated normal cognition. GENDER, OCTO‐Twin, and SATSA are longitudinal cohort studies. All of these twins underwent cognitive assessment at entry and at either 2‐year intervals (OCTO‐Twin and later waves of SATSA), 3‐year intervals (earlier waves of SATSA), or 4‐year intervals (GENDER) over the course of their follow‐up (telephone cognitive screening if they missed a follow‐up in the longitudinal wave). Cognitive assessment for SATSA encompassed information, synonyms, analogies, figure logic, block design, card rotations, digit span, picture memory, names and faces, digit symbol, figure identification, and word list immediate and delayed recall, with the others designed to be parallel.^26^ Participants were referred for further dementia work‐up analogous to HARMONY if their cognitive assessment or telephone screening evidenced impairments, with diagnoses assigned by a consensus panel. Twins who received a diagnosis of dementia had their age at onset established through integrating any available data from informant reports, medical records, and longitudinal cognitive trajectories.

All twins continued to be followed through a linkage to the population‐based registries, including the National Patient Register (inpatient and outpatient specialist care records), the Cause of Death Register, and the Prescribed Drug Register. These registries document International Classification of Disease (ICD) codes for primary and additional diseases or underlying and contributing causes of death, or Anatomical Therapeutic Chemical (ATC) codes for dispensed medication, including codes indicating a dementia diagnosis. Twins were excluded from the control group if an ICD or ATC code indicating dementia appeared in the registries subsequent to their initial evaluation for dementia.^27^

#### 2.2.2 Mortality and cause of death

For twins who are deceased, information on the date and cause of death was obtained from the Cause of Death Register. Data used in the present study were updated to December 31, 2016. Cause of death was compared between MZ cases and their co‐twin controls as part of post hoc analysis to examine any clear pattern in their cause of death that might explain the results we found.

#### 2.2.3 Covariates

We included age at dementia onset, sex, self‐rated health, and educational attainment as covariates in the statistical models. *Age at dementia onset* for the case twin was used as the timepoint to start measuring the number of years survived (see Section 2.3, Statistical analysis) and included in the models to adjust for any differences in the underlying time scale depending on the person's age. For instance, 1 year of survival may not have the same weight on the mortality risk for individuals in their 60s versus those in their 80s. Age at dementia onset was established retrospectively by a detailed report of onset and course of symptoms.^28^ For individuals who were assessed longitudinally, age of onset was the point between the age when assessed as cognitively normal or with mild cognitive impairment and the age at which dementia was established. *Sex* was included to account for sex differences in mortality (i.e., women live longer than men). *Self‐rated health* assesses individuals’ perception of their own health, providing a marker of physical and mental functioning, health behaviors, the presence of illness, and mortality.^29^, ^30^, ^31^ Self‐rated health was based on some version of “How would you rate your overall health?” with the scoring harmonized across different answer categories from the different studies.^32^ Both individuals and informants were asked the question during dementia evaluations. In the present study, self‐rated health data were converted to T‐scores. Higher scores indicate worse health status perceived by the individual. Studies have consistently shown that higher levels of education predict attenuated risk for mortality.^33^ *Education* was coded according to the International Standard Classification of Education (ISCED) and defined as the highest ISCED level completed by the individual,^34^ which ranges from 0 (less than primary education) to 8 (doctoral or equivalent level). Educational attainment was asked in each study including either highest level achieved and/or number of years of education. Where the question was asked on more than one occasion to the same individual, all sources of information were used. Coders translated these answers into ISCED categories. ISCED documentation includes country‐specific mapping to aid in coding.^34^

### 2.3 Statistical analysis

Life tables analyses were performed using SAS 9.4 (Cary, NC), and random effects Cox regressions were conducted using Mplus 8.8 (Muthén & Muthén, 1998‐2022). In all survival analyses, the criterion variable was the number of years since the time of the case's dementia onset until death or right‐censoring. For deceased twins, time to death was calculated by subtracting the case's age at onset from their age at death. For individuals who were right‐censored (ie, those who were alive at the end of follow‐up), the time to censoring was computed based on subtracting the case's age at onset from their age as of December 31, 2016.

#### 2.3.1 Case‐control and co‐twin control analyses

Independent samples *t*‐tests and chi‐square tests were used to describe demographic characteristics of cases and controls. Life tables were generated to compare survival function and median survival time since the case's age at dementia onset between cases and controls. We used multilevel continuous‐time survival analysis using Cox regression to estimate mortality risk associated with dementia diagnosis within‐cluster (i.e., case‐matched controls and discordant pairs). This approach estimates a random effect to adjust for within‐cluster homogeneity and estimates hazard ratios (HRs) that adjust for shared confounds within twin pairs.^35^ This approach is preferable to stratified Cox regression models, as it provides a more robust test of within‐clustering effects in twins.^35^ Mortality risk was treated as a semi‐parametric survival variable so that full information maximum likelihood could be used for the covariates in unadjusted and adjusted models. In Mplus, semi‐parametric Cox regression models use as a baseline hazard function a stepwise function that approximates the non‐parametric Cox regression stepwise function. This value is set to 10, but in order to improve precision of the baseline hazard function, we set the value to 50. Full information maximum likelihood was used to estimate all parameter estimates. In the case‐control analysis, sex and age at dementia onset were included to adjust for between‐cluster effects of each covariate on mortality risk.

#### 2.3.2 Post hoc analysis

Demographic characteristics of MZ co‐twin controls and unrelated MZ controls were examined using independent samples *t*‐tests and chi‐square tests. Life tables were constructed to describe the survival function and median survival time since the MZ case's age at dementia onset for MZ co‐twin controls and unrelated MZ controls. Multilevel continuous‐time survival analysis using Cox regression was conducted to evaluate mortality risk of MZ co‐twin controls and unrelated MZ controls, adjusting for covariates.

## 3 RESULTS

Table 1 presents characteristics of the sample included in the case‐control analysis. Sample characteristics of the co‐twin control models and post hoc analysis and results from sensitivity analysis are included in the supplemental materials. Table 2 shows HRs and 95% confidence intervals (CIs) of the unadjusted and adjusted survival models from case‐control and co‐twin control analyses, as well as post hoc analysis.

**TABLE 2:** Hazard ratios and 95% confidence intervals of the case‐control, co‐twin control, and post hoc analyses.

Design		Unadjusted Model	Adjusted Model
Case‐control	Case	**2.09** (1.88, 2.22)	**2.02** (1.86, 2.18)
	Age at dementia onset		**3.78** (3.57, 4.01)
	Female		**0.61** (0.56, 0.65)
	Education^a^		**0.92** (0.89, 0.95)
	Self‐rated health^b^		**1.04** (1.03, 1.05)
	Residual variance	0.91 (0.78, 1.03)	0.06 (0.04, 0.08)
DZ co‐twin control	Case	**1.55** (1.28, 1.87)	**1.67** (1.39, 2.00)
	Age at dementia onset		**2.88** (2.48, 3.33)
	Female		**0.58** (0.48, 0.70)
	Education		0.94 (0.85, 1.04)
	Self‐rated health		**1.17** (1.06, 1.30)
	Residual variance	0.52 (0.23, 0.81)	0.08 (0.03, 0.13)
MZ co‐twin control	Case	1.27 (0.91, 1.77)	**1.48** (1.08, 2.04)
	Age at dementia onset		**3.26** (2.43, 4.38)
	Female	NA	NA
	Education		0.96 (0.83, 1.11)
	Self‐rated health		1.19 (0.99, 1.43)
	Residual Variance	0.83 (0.21, 1.47)	0.10 (−0.02, 0.21)
MZ co‐twin controls	MZ Co‐twin Controls	**1.40** (1.07, 1.84)	**1.32** (1.03, 1.70)
vs Unrelated	Age at dementia onset		**3.69** (3.24, 4.20)
MZ controls	Female	NA	NA
(post hoc)	Education		0.97 (0.89, 1.05)
	Self‐rated health		1.13 (1.00, 1.27)
	Residual variances	0.86 (0.42, 1.31)	0.00 (0.01, 0.02)

*Note*: Hazard ratios in bold are statistically significant at *p* < 0.05, two‐tailed.

Abbreviations: DZ, dizygotic; MZ, monozygotic.

^a^ International Standard Classification of Education (ISCED) codes.

^b^ Data are shown in T‐scores (mean = 50, SD = 10).

### 3.1 Case‐control analysis

Cases had lower levels of education than controls (*p* < 0.001). There was no difference between cases and controls in self‐rated health (*p* = 0.37). A higher proportion of cases were deceased compared to controls (*p* < 0.001). Among those who died, the mean number of years lived since case's age at dementia onset was lower for cases than controls (*p* < 0.001) (Table 1).

**FIGURE 2:**
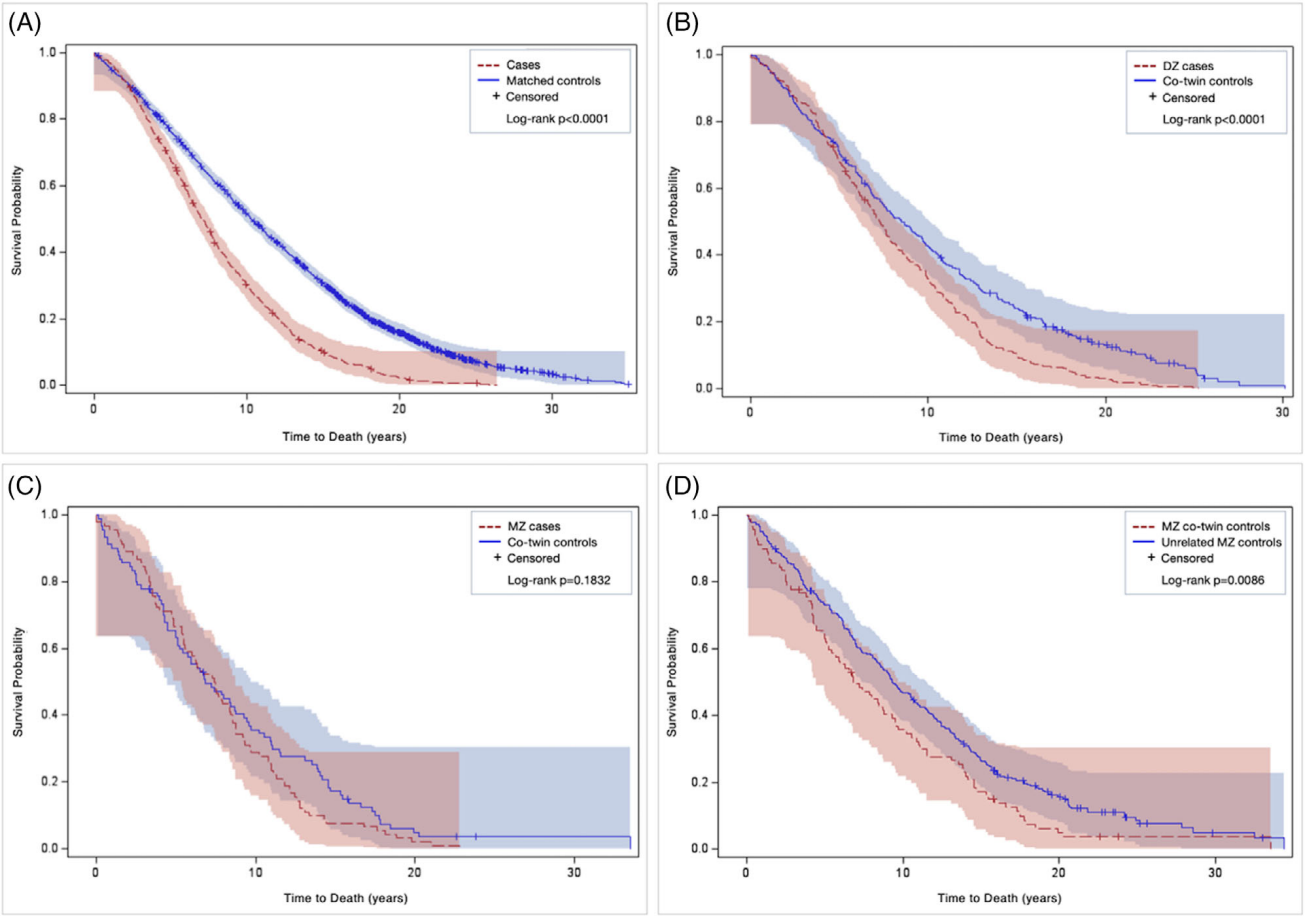
Survival functions. Kaplan‐Meier survival functions of time to death or right‐censoring contrasting cases and matched controls in the total sample (A), DZ cases and their co‐twin controls (B), MZ cases and their co‐twin controls (C), and MZ co‐twin controls and unrelated MZ controls matched on age and sex (D). Shaded areas show 95% confidence intervals. DZ, dizygotic; MZ, monozygotic.

Figure 2A shows Kaplan‐Meier survival curves for cases and controls. These results are unadjusted for covariates and within‐pair clustering. Median survival estimates indicated 7.06 years for cases (95% CI: 6.79, 7.32) and 10.25 years for controls (95% CI: 10.06, 10.52) (Log‐rank chi‐square = 252.77, *p* < 0.001). Kaplan‐Meier results showed that cases had a twofold increase in the mortality risk compared with controls (Table 2).

### 3.2 Co‐twin control analyses

#### 3.2.1 DZ co‐twin control

Compared to their co‐twins without dementia, a greater proportion of the cases were deceased (*p* < 0.0001). There were no statistically significant differences between cases and their co‐twin controls in their mean number of years lived (when both were deceased) (*p* = 0.09), levels of education (*p* = 0.24), and self‐rated health (*p* = 0.71) (Table S1). Figure 2B shows Kaplan‐Meier survival curves for cases and their co‐twin controls in DZ discordant pairs. Median survival time was estimated to be 7.32 years (95% CI: 6.92, 7.65) for cases and 8.04 years (95% CI: 7.45, 8.76) for their co‐twin controls, and there was a significant difference between these estimates (Log‐rank chi‐square = 20.71, *p* < 0.001). Mortality risk was attenuated in DZ pairs discordant for dementia, compared to the total sample of cases and matched controls, with 1.67 times greater risk for mortality in DZ cases compared to their co‐twin controls, adjusting for effects of age at dementia diagnosis, sex, education, and self‐rated health (Table 2). As a sensitivity analysis, we divided the DZ sample by sex using same‐sex twins only and found that DZ male and female cases both had significantly greater risk of dementia than co‐twin controls, although the effect was larger in female pairs than in male pairs (Table S2).

#### 3.2.2 MZ co‐twin control

A higher proportion of the cases were deceased than their co‐twin controls (*p* = 0.02). There were no statistically significant differences between cases and their co‐twin controls in their mean number of years lived (when both were deceased) (*p* = 0.73), education (*p* = 0.63), and self‐rated health (*p* = 0.95) (Supplemental Table S3). Figure 2C presents Kaplan‐Meier survival curves for cases and their co‐twin controls in MZ discordant pairs. There was no statistically significant difference between cases (7.45 years, 95% CI: 6.21, 7.93) and their co‐twin controls (6.85 years, 95% CI: 5.92, 8.44) in the median survival time (Log‐rank chi‐square = 1.77, *p* = 0.18). Holding shared genetic and environmental factors constant, MZ cases nevertheless were 1.48 times more likely to die following diagnosis than their undiagnosed co‐twins. This result indicates a statistically significant difference between cases and their co‐twin controls, adjusting for effects of age at dementia diagnosis, education, and self‐rated health (Table 2). As in the DZ analysis above, we divided the sample by sex and found that only MZ female cases had significantly greater risk of dementia than co‐twin controls (Table S2).

### 3.3 Post hoc analysis

MZ co‐twin controls (ie, unaffected co‐twins of MZ cases) did not differ from unrelated MZ controls (ie, individuals from MZ pairs where neither twin had dementia) in the proportion of deceased twins (*p* = 0.11), mean number of years lived (when both were deceased) (*p* = 0.11), levels of education (*p* = 0.19), and self‐rated health (*p* = 0.51) (Table S4). Figure 2D presents Kaplan‐Meier survival curves for MZ co‐twin controls and unrelated MZ controls. Again, these estimates were unadjusted for covariates or within‐pair clustering. In this model, median survival time was 9.30 years (95% CI: 8.70, 10.50) for unrelated MZ controls, which was significantly greater than 6.85 years for MZ co‐twin controls (Log‐rank chi‐square = 6.91, *p* < 0.01). Results from survival analysis showed that MZ co‐twin controls had 1.32 times greater risk for mortality than unrelated MZ twins, which was statistically significant after adjusting for education, self‐rated health, and age that the MZ case (ie, co‐twin control's twin) was diagnosed with dementia (Table 2).

MZ co‐twin controls were further examined for their primary cause of death, stratified by their time of death in relation to the case's death. The frequencies of cause of death categories (eg, cardiovascular disease, cancer) are presented in Supplemental Table S5. There was no clear pattern distinguishing the MZ co‐twin controls who died before or within 1 year of the case's death and those who outlived the cases by more than a year.

## 4 DISCUSSION

We sought to estimate the association between dementia and mortality in older adults using case‐control and co‐twin control models. As expected, in the case‐control analysis, similar in design to extant literature on survival after dementia diagnosis, individuals with dementia had significantly higher risk of death compared with their age‐ and sex‐matched peers. This finding is consistent with previous studies that reported that dementia is related to increased risk of death.^1^, ^3^ Our estimate of 7 years median survival since age at dementia onset is comparable to 8.3 years median survival for cases with onset at age 65 in the Baltimore Longitudinal Study of Aging cohort^3^ or 5.8 years mean survival for Alzheimer's disease (AD) cases from a recent large meta‐analysis.^1^ It is also consistent with an average survival of 4 to 8 years after a diagnosis of AD, summarized in the 2022 annual report of the Alzheimer's Association.^36^

In DZ twin pairs discordant for dementia diagnosis, we note that the magnitude of the difference in survival between cases and their co‐twin controls was attenuated relative to the effect observed in the case‐control analysis, though it remained elevated. As hypothesized, in MZ twins discordant for dementia, this elevated risk for mortality was further attenuated but remained statistically significant. Sensitivity analyses showed attenuation for males such that there was no longer a significantly elevated risk for mortality, but there was relatively little attenuation for female MZ pairs compared to female DZ pairs. This pattern of results showing proportional decreases in risk estimates in DZ and MZ pairs discordant for dementia compared to the total sample suggests that both genetic and environmental factors in common to twins within pairs explain the association between dementia onset and mortality. The difference in effects between the case‐control sample and the discordant DZ sample was larger than the difference between the discordant DZ and discordant MZ sample. In this way, shared environmental variance may be a larger confound than shared genetic variance. The smaller HR in the MZ twins compared to the DZ twins further suggests genetic confounding, especially in male twins. At the same time, the significantly increased risk of mortality in MZ cases compared to their undiagnosed co‐twins reflects environmental factors unique to each twin, accounting for the association between dementia diagnosis and mortality risk.

Subsequent post hoc analysis found that MZ controls (ie, undiagnosed twins whose co‐twin had dementia) had elevated mortality risk compared to unrelated MZ controls from pairs in which neither twin was diagnosed with dementia. Excess mortality risk in the MZ controls further supports the conclusion that genetic variance contributes to the association between dementia risk and mortality. In other words, even though identical twins with co‐twins affected by dementia may never suffer from dementia themselves, they, too, may have a shortened lifespan. Median survival time for MZ controls whose co‐twin had dementia was comparable to the cases in the case‐control analysis, while median survival time for the unrelated MZ controls whose co‐twin was dementia‐free was comparable to the controls in the case‐control analysis.

Taken together, these results suggest that genetic factors confound the association between dementia and mortality. Given that genes have significant effects on the etiology of AD and other dementias,^37^, ^38^ it is reasonable to infer that some of the same genetic influences associated with dementia may also affect longevity. Previous research data suggest that apolipoprotein E (*APOE*) may be the primary candidate gene among those shared by dementia and mortality, as it is strongly associated with incident dementia and increased mortality.^39^, ^40^, ^41^ In summary, the case‐control study confirmed that dementia is associated with elevated risk for mortality. In turn, the co‐twin control models, corroborated by the post hoc test, suggest that the association between dementia diagnosis and increased mortality is explained in part by genetic and environmental factors shared by twins in the same family. Co‐twin controls, like their co‐twins with dementia, may have shortened lifespans by virtue of their genotype when compared to unaffected families. Nonetheless, after accounting for shared influences and other covariates, a significant association remains between dementia and elevated risk for mortality. In MZ pairs discordant for dementia, environmental factors (eg, differences in education, occupation, child and adult family life, and other lifestyle choices) unique to the undiagnosed co‐twin could account for the absence of a dementia diagnosis. In turn, the magnitude of the difference in mortality risk for the MZ twin with dementia may provide an indication of how much the disease itself may shorten the lifespan.

There are limitations to consider. First, while the current study employed a population‐based sample of twins who underwent a comprehensive dementia workup and were followed through death by clinical visits and linkage to national registries, participants are comprised of Swedish twins, and our results may not be generalized to other samples. The comparability of our case‐control results to prior reports of effects of dementia on mortality are reassuring in this respect. Second, our results combine all dementias, although different types of dementia are known to be associated with different effects on mortality.^1^ Sample sizes precluded focusing on AD alone. However, the bulk of cases are AD, and previous work with the STR has shown that results for AD parallel those for all dementias.^8^, ^42^ Although this is the largest twin study of dementia and mortality risk to date, we encourage other large twin registries to replicate our findings. Future studies may help elucidate specific genetic and shared environmental factors that underpin the risk shared by dementia and mortality.

To our knowledge, the current study is the first to examine the relationship between dementia and mortality using the genetically informative twin method. The results have important practical implications for individuals diagnosed with dementia and their families. The estimates of survival time since dementia onset based on this study offer further information that may be helpful for patients, families, healthcare providers, and public health policymakers to develop care plans, gauge costs, and make financial decisions. Families tend to experience less stress as they have more discussions about end‐of‐life decisions and advanced care planning, despite the difficulty of having such discussions.^43^ Finally, our key finding from the co‐twin control models provides new insight into the mechanisms underlying the association between dementia and increased mortality.

## CONFLICT OF INTEREST STATEMENT

Jung Yun Jang and Christopher R. Beam report no disclosures. Ida K. Karlsson received support from The Nordic Gerontological Federation Promising Researcher Award and Mobility Grant from Eurolife. Nancy L. Pedersen received support from NIH Grant No. R01 AG059329. Margaret Gatz received support from NIH Grant Nos. R01 AG059329 and R01 AG054442 and is a member of Health and Research Advisory Council of Alzheimer's Los Angeles (no financial relationship). Author disclosures are available in the supporting information.

## CONSENT STATEMENT

All participants provided informed consent.

## Supporting information

Supporting Information

Supporting Information
