# Supplementary material for: Dementia and mortality in older adults: A twin study
Source: Alzheimers Dement. 2023 Dec 11;20(3):1682–92. doi: 10.1002/alz.13553 (PMC10947969; doi:10.1002/alz.13553)
Supplement: Supplementary file 1 — Supporting Information [file ALZ-20-1682-s001.docx]

Supplemental Table S1. Sample characteristics for cases and co-twin controls in DZ co-twin control analysis

|  |  | Cases  (n = 288) | Controls  (n = 288) | t or χ^2^ | *P* |
| --- | --- | --- | --- | --- | --- |
| Birth year | range | 1899 - 1937 | 1899 - 1937 | -- | -- |
| Female | n (%) | 176 (61.1) | 172 (59.7) | 0.12 | 0.73 |
| Age at dementia onset | mean (SD) | 77.47 (6.99) | -- | -- | -- |
| Education* | mean (SD) | 1.37 (0.83) | 1.46 (0.99) | 1.17 | 0.24 |
| Self-rated health† | mean (SD) | 54.90 (11.15) | 54.49 (10.46) | -0.37 | 0.71 |
| Deceased | n (%) | 285 (99.0) | 262 (91.0) | 19.21 | <.0001 |
| Time to death‡ | mean (SD) | 8.10 (5.13) | 8.94 (6.36) | 1.71 | 0.09 |

Abbreviation: DZ, dizygotic

*International Standard Classification of Education (ISCED) codes

†Data are shown in T-scores (mean = 50, SD = 10).

‡In each case and co-twin control cluster, time to death was calculated based on age at death − case twin’s age at dementia onset, for twins who are deceased.

Supplemental Table S2. Hazard ratios and 95% confidence intervals of the co-twin control analyses by sex

| Design |  | Unadjusted Model | Adjusted Model |
| --- | --- | --- | --- |
| DZ male | Case | 1.23 (0.83, 1.84) | **1.49** (1.02, 2.19) |
|  | Age at dementia onset |  | **3.75** (2.73, 5.16) |
|  | Education* |  | 0.99 (0.79, 1.23) |
|  | Self-rated health† |  | 1.24 (0.97, 1.59) |
| ‍ | Residual variance | 0.60 (0.003, 1.20) | 0.01^‡^ |
| DZ female | Case | **1.62** (1.22, 2.15) | **1.73** (1.32, 2.26) |
|  | Age at dementia onset |  | **2.54** (2.19, 2.94) |
|  | Education |  | 0.98 (0.83, 1.16) |
|  | Self-rated health |  | **1.20** (1.05, 1.38) |
| ‍ | Residual variance | 0.46 (0.14, 0.79) | 0.01^‡^ |
| MZ male | Case | 1.00 (0.62, 1.61) | 1.19 (0.42, 3.38) |
|  | Age at dementia onset |  | 2.88 (0.02, 362.96) |
|  | Education |  | 0.96 (0.36, 2.53) |
|  | Self-rated health |  | 1.10 (0.82, 1.48) |
| ‍ | Residual Variance | 0.74 (-0.09, 1.56) | 0.16 (-2.73, 2.60) |
| MZ female | Case | 1.45 (0.92, 2.30) | **1.71** (1.09, 2.70) |
|  | Age at dementia onset |  | **3.57** (2.25, 5.66) |
|  | Education |  | 0.97 (0.80, 1.18) |
|  | Self-rated health |  | 1.27 (0.99, 1.64) |
| ‍ | Residual variances | 0.67 (-0.08, 1.41) | 0.01^‡^ |

Abbreviations: DZ, dizygotic, MZ, monozygotic

Hazard ratios in bold are statistically significant at *P* < .05, two-tailed.

*International Standard Classification of Education (ISCED) codes

†Data are shown in T-scores (mean = 50, SD = 10).

‡ Values fixed to facilitate model convergence.

Supplemental Table S3. Sample characteristics for cases and co-twin controls in MZ co-twin control analysis

|  |  | Cases  (n = 90) | Controls  (n = 90) | t or χ^2^ | *P* |
| --- | --- | --- | --- | --- | --- |
| Birth year | range | 1900 - 1931 | 1900 - 1931 | -- | -- |
| Female | n (%) | 48 (53.3) | 48 (53.3) | -- | -- |
| Age at dementia onset | mean (SD) | 79.18 (7.64) | -- | -- | -- |
| Education* | mean (SD) | 1.46 (1.10) | 1.54 (1.25) | 0.48 | 0.63 |
| Self-rated health† | mean (SD) | 53.23 (11.08) | 53.35 (10.39) | 0.06 | 0.95 |
| Deceased | n (%) | 90 (100.0) | 85 (94.4) | 5.14 | 0.02 |
| Time to death‡ | mean (SD) | 7.78 (4.99) | 8.08 (6.17) | 0.35 | 0.73 |

Abbreviation: MZ, monozygotic

*International Standard Classification of Education (ISCED) codes

†Data are shown in T-scores (mean = 50, SD = 10).

‡In each case and co-twin control cluster, time to death was calculated based on age at death − case twin’s age at dementia onset, for twins who are deceased.

Supplemental Table S4. Sample characteristics for MZ co-twin controls and unrelated MZ controls in *post hoc* analysis

|  |  | MZ controls  (n = 90) | Unrelated MZ controls | t or χ^2^ | *P* |
| --- | --- | --- | --- | --- | --- |
|  |  |  | (n = 264) |  |  |
| Birth year | range | 1900 - 1931 | 1900 – 1932 | -- | -- |
| Female | n (%) | 48 (53.3) | 144 (54.6) | 0.04 | 0.84 |
| Education* | mean (SD) | 1.54 (1.25) | 1.76 (1.38) | 1.31 | 0.19 |
| Self-rated health† | mean (SD) | 53.35 (10.39) | 52.48 (9.87) | -0.66 | 0.51 |
| Deceased | n (%) | 85 (94.4) | 234 (88.6) | 2.54 | 0.11 |
| Time to death‡ | mean (SD) | 8.08 (6.17) | 9.41 (6.58) | 1.62 | 0.11 |

Abbreviation: MZ, monozygotic

*International Standard Classification of Education (ISCED) codes

†Data are shown in T-scores (mean = 50, SD = 10).

‡In each cluster of MZ co-twin control and unrelated MZ controls, time to death was calculated based on age at death − MZ case twin’s age at dementia onset, for twins who are deceased.

Supplemental Table S5. Frequencies (column percentages) of primary cause of death for MZ co-twin controls

| Cause of death | MZ co-twin controls died first or within 1 year of case’s death | MZ co-twin controls outlived case > 1 year |
| --- | --- | --- |
| Cardiovascular disease | 15 (34.1) | 18 (45.0) |
| Cerebrovascular disease | 3 (6.8) | 5 (12.5) |
| Cancer | 11 (25.0) | 8 (12.5) |
| Pulmonary disease | 2 (4.6) | 0 (0.0) |
| Infections | 4 (9.1) | 6 (15.0) |
| Parkinson’s disease | 1 (2.3) | 0 (0.0) |
| Other organ failure/dysfunction | 3 (6.8) | 3 (7.5) |
| Accidents/falls | 4 (9.1) | 1 (2.5) |
| Senility | 0 (0.0) | 2 (5.0) |
| Other/unknown | 1 (2.3) | 0 (0.0) |
| Total | 44 | 40 |

Abbreviation: MZ, monozygotic
