# Supplementary material for: Dementia and mortality in older adults: A twin study
Source: Alzheimers Dement. 2023 Dec 11;20(3):1682–92. doi: 10.1002/alz.13553 (PMC10947969; doi:10.1002/alz.13553)
Supplement: Supplementary file 2 — Supporting Information [file ALZ-20-1682-s002.pdf]

ICMJE DISCLOSURE FORM

Date: 9/19/2023

Your Name: Jung Yun Jang

Manuscript Title: Dementia and Mortality: A Twin Study

Manuscript Number (if known): ADJ-D-23-00711

In the interest of transparency, we ask you to disclose all relationships/activities/interests listed below that are related to the content of your manuscript. "Related" means any relation with for-profit or not-for-profit third parties whose interests may be affected by the content of the manuscript. Disclosure represents a commitment to transparency and does not necessarily indicate a bias. If you are in doubt about whether to list a relationship/activity/interest, it is preferable that you do so.

The author's relationships/activities/interests should be defined broadly. For example, if your manuscript pertains to the epidemiology of hypertension, you should declare all relationships with manufacturers of antihypertensive medication, even if that medication is not mentioned in the manuscript.

In item #1 below, report all support for the work reported in this manuscript without time limit. For all other items, the time frame for disclosure is the past 36 months.

		**Name all entities with whom you have this relationship or indicate none (add rows as needed)**	**Specifications/Comments (e.g., if payments were made to you or to your institution)**
**Time frame: Since the initial planning of the work**			
**1**	All support for the present manuscript (e.g., funding, provision of study materials, medical writing, article processing charges, etc.) **No time limit for this item.**	☒ None	
			Click the tab key to add additional rows.
**Time frame: past 36 months**			
**2**	Grants or contracts from any entity (if not indicated in item #1 above).	☒ **None**	
**3**	Royalties or licenses	☒ **None**	

		Name all entities with whom you have this relationship or indicate none (add rows as needed)	Specifications/Comments (e.g., if payments were made to you or to your institution)
**4**	Consulting fees	☒ **None**	
**5**	Payment or honoraria for lectures, presentations, speakers bureaus, manuscript writing or educational events	☒ **None**	
**6**	Payment for expert testimony	☒ **None**	
**7**	Support for attending meetings and/or travel	☒ **None**	
**8**	Patents planned, issued or pending	☒ **None**	
**9**	Participation on a Data Safety Monitoring Board or Advisory Board	☒ **None**	
**10**	Leadership or fiduciary role in other board, society, committee or advocacy group, paid or unpaid	☒ **None**	

		Name all entities with whom you have this relationship or indicate none (add rows as needed)	Specifications/Comments (e.g., if payments were made to you or to your institution)
**11**	Stock or stock options	☒ **None**	
**12**	Receipt of equipment, materials, drugs, medical writing, gifts or other services	☒ **None**	
**13**	Other financial or non-financial interests	☒ **None**	

Please place an "X" next to the following statement to indicate your agreement:

☒ I certify that I have answered every question and have not altered the wording of any of the questions on this form.

ICMJE DISCLOSURE FORM

Date: C9/18/2023

Your Name: Christopher R. Beam

Manuscript Title: Dementia and Mortality in Older Adults: A Twin Study

Manuscript Number (if known): ADJ-D-23-00711

In the interest of transparency, we ask you to disclose all relationships/activities/interests listed below that are related to the content of your manuscript. "Related" means any relation with for-profit or not-for-profit third parties whose interests may be affected by the content of the manuscript. Disclosure represents a commitment to transparency and does not necessarily indicate a bias. If you are in doubt about whether to list a relationship/activity/interest, it is preferable that you do so.

The author's relationships/activities/interests should be defined broadly. For example, if your manuscript pertains to the epidemiology of hypertension, you should declare all relationships with manufacturers of antihypertensive medication, even if that medication is not mentioned in the manuscript.

In item #1 below, report all support for the work reported in this manuscript without time limit. For all other items, the time frame for disclosure is the past 36 months.

		Name all entities with whom you have this relationship or indicate none (add rows as needed)	Specifications/Comments (e.g., if payments were made to you or to your institution)
**Time frame: Since the initial planning of the work**			
1	All support for the present manuscript (e.g., funding, provision of study materials, medical writing, article processing charges, etc.) **No time limit for this item.**	None	
		National Institute on Aging (NIH grant #: R01 AG060470)	Research effort
		National Institute on Aging (NIH grant #: T32 AG000037)	Post-doctoral trainee
			Click the tab key to add additional rows.
**Time frame: past 36 months**			
**2**	Grants or contracts from any entity (if not indicated in item #1 above).	X **None**	
**3**	Royalties or licenses	X **None**	

		Name all entities with whom you have this relationship or indicate none (add rows as needed)	Specifications/Comments (e.g., if payments were made to you or to your institution)
**4**	Consulting fees	X **None**	
**5**	Payment or honoraria for lectures, presentations, speakers bureaus, manuscript writing or educational events	X **None**	
**6**	Payment for expert testimony	X **None**	
**7**	Support for attending meetings and/or travel	X **None**	
**8**	Patents planned, issued or pending	X **None**	
**9**	Participation on a Data Safety Monitoring Board or Advisory Board	X **None**	
**10**	Leadership or fiduciary role in other board, society, committee or advocacy group, paid or unpaid	X **None**	

		Name all entities with whom you have this relationship or indicate none (add rows as needed)	Specifications/Comments (e.g., if payments were made to you or to your institution)
**11**	Stock or stock options	X **None**	
**12**	Receipt of equipment, materials, drugs, medical writing, gifts or other services	X **None**	
**13**	Other financial or non-financial interests	X **None**	

Please place an "X" next to the following statement to indicate your agreement:

X I certify that I have answered every question and have not altered the wording of any of the questions on this form.

ICMJE DISCLOSURE FORM

**Date:**	9/18/2023
**Your Name:**	Ida Karlsson
**Manuscript Title:**	Dementia and Mortality in Older Adults: A Twin Study
**Manuscript Number (if known):**	ADJ-D-23-00711

In the interest of transparency, we ask you to disclose all relationships/activities/interests listed below that are related to the content of your manuscript. "Related" means any relation with for-profit or not-for-profit third parties whose interests may be affected by the content of the manuscript. Disclosure represents a commitment to transparency and does not necessarily indicate a bias. If you are in doubt about whether to list a relationship/activity/interest, it is preferable that you do so.

The author's relationships/activities/interests should be defined broadly. For example, if your manuscript pertains to the epidemiology of hypertension, you should declare all relationships with manufacturers of antihypertensive medication, even if that medication is not mentioned in the manuscript.

In item #1 below, report all support for the work reported in this manuscript without time limit. For all other items, the time frame for disclosure is the past 36 months.

		**Name all entities with whom you have this relationship or indicate none (add rows as needed)**	**Specifications/Comments (e.g., if payments were made to you or to your institution)**
**Time frame: Since the initial planning of the work**			
1	All support for the present manuscript (e.g., funding, provision of study materials, medical writing, article processing charges, etc.) **No time limit for this item.**	☐ **None**	
		The Swedish Research Council for Health, Working Life and Welfare	Payment to my institution
		The Strategic Research Program in Epidemiology at Karolinska Institutet	Payment to my institution
		Loo and Hans Osterman's Foundation	Payment to my institution
		Foundation for Geriatric Diseases at Karolinska Institutet	Payment to my institution
		Karolinska Institutet's Research Foundation	Payment to my institution
**Time frame: past 36 months**			
2	Grants or contracts from any entity (if not indicated in item #1 above).	☐ **None**	
		The Nordic Gerontological Federation	Promising Researcher award, 20.000 SEK (approximately 1672 EUR or 1784 USD), paid to me
3	Royalties or licenses	☒ **None**	

		Name all entities with whom you have this relationship or indicate none (add rows as needed)	Specifications/Comments (e.g., if payments were made to you or to your institution)
**4**	Consulting fees	☒ **None**	
**5**	Payment or honoraria for lectures, presentations, speakers bureaus, manuscript writing or educational events	☒ **None**	
**6**	Payment for expert testimony	☒ **None**	
**7**	Support for attending meetings and/or travel	☐ **None**	
		Eurolife	Mobility grant, paid to my institution (1500 EUR)
**8**	Patents planned, issued or pending	☒ **None**	
**9**	Participation on a Data Safety Monitoring Board or Advisory Board	☒ **None**	
**10**	Leadership or fiduciary role in other board, society, committee or advocacy group, paid or unpaid	☒ **None**	

		Name all entities with whom you have this relationship or indicate none (add rows as needed)	Specifications/Comments (e.g., if payments were made to you or to your institution)
**11**	Stock or stock options	☒ **None**	
**12**	Receipt of equipment, materials, drugs, medical writing, gifts or other services	☒ **None**	
**13**	Other financial or non-financial interests	☒ **None**	

Please place an "X" next to the following statement to indicate your agreement:

☒ I certify that I have answered every question and have not altered the wording of any of the questions on this form.

ICMJE DISCLOSURE FORM

**Date:**	9/18/2023
**Your Name:**	Nancy L. Pedersen
**Manuscript Title:**	Dementia and Mortality in Older Adults: A Twin Study
**Manuscript Number (if known):**	ADJ-D-23-00711

In the interest of transparency, we ask you to disclose all relationships/activities/interests listed below that are related to the content of your manuscript. "Related" means any relation with for-profit or not-for-profit third parties whose interests may be affected by the content of the manuscript. Disclosure represents a commitment to transparency and does not necessarily indicate a bias. If you are in doubt about whether to list a relationship/activity/interest, it is preferable that you do so.

The author's relationships/activities/interests should be defined broadly. For example, if your manuscript pertains to the epidemiology of hypertension, you should declare all relationships with manufacturers of antihypertensive medication, even if that medication is not mentioned in the manuscript.

In item #1 below, report all support for the work reported in this manuscript without time limit. For all other items, the time frame for disclosure is the past 36 months.

		**Name all entities with whom you have this relationship or indicate none (add rows as needed)**	**Specifications/Comments (e.g., if payments were made to you or to your institution)**
		Time frame: Since the initial planning of the work	
1	All support for the present manuscript (e.g., funding, provision of study materials, medical writing, article processing charges, etc.) **No time limit for this item.**	☐ None	
		NIH Grant No. R01 AG060470	Grant provided to my institution
			Click the tab key to add additional rows.
		Time frame: past 36 months	
2	Grants or contracts from any entity (if not indicated in item #1 above).	☐ **None**	
		NIH Grant No. R01 AG059329	Grant provided to my institution
3	Royalties or licenses	☒ **None**	

		Name all entities with whom you have this relationship or indicate none (add rows as needed)	Specifications/Comments (e.g., if payments were made to you or to your institution)
**4**	Consulting fees	☒ **None**	
**5**	Payment or honoraria for lectures, presentations, speakers bureaus, manuscript writing or educational events	☒ **None**	
**6**	Payment for expert testimony	☒ **None**	
**7**	Support for attending meetings and/or travel	☒ **None**	
**8**	Patents planned, issued or pending	☒ **None**	
**9**	Participation on a Data Safety Monitoring Board or Advisory Board	☒ **None**	
**10**	Leadership or fiduciary role in other board, society, committee or advocacy group, paid or unpaid	☒ **None**	

		Name all entities with whom you have this relationship or indicate none (add rows as needed)	Specifications/Comments (e.g., if payments were made to you or to your institution)
**11**	Stock or stock options	☒ **None**	
**12**	Receipt of equipment, materials, drugs, medical writing, gifts or other services	☒ **None**	
**13**	Other financial or non-financial interests	☒ **None**	

Please place an "X" next to the following statement to indicate your agreement:

☒ I certify that I have answered every question and have not altered the wording of any of the questions on this form.

ICMJE DISCLOSURE FORM

**Date:**	9/18/2023
**Your Name:**	Margaret Gatz
**Manuscript Title:**	Dementia and Mortality in Older Adults: A Twin Study
**Manuscript Number (if known):**	ADJ-D-23-00711

In the interest of transparency, we ask you to disclose all relationships/activities/interests listed below that are related to the content of your manuscript. "Related" means any relation with for-profit or not-for-profit third parties whose interests may be affected by the content of the manuscript. Disclosure represents a commitment to transparency and does not necessarily indicate a bias. If you are in doubt about whether to list a relationship/activity/interest, it is preferable that you do so.

The author's relationships/activities/interests should be defined broadly. For example, if your manuscript pertains to the epidemiology of hypertension, you should declare all relationships with manufacturers of antihypertensive medication, even if that medication is not mentioned in the manuscript.

In item #1 below, report all support for the work reported in this manuscript without time limit. For all other items, the time frame for disclosure is the past 36 months.

		**Name all entities with whom you have this relationship or indicate none (add rows as needed)**	**Specifications/Comments (e.g., if payments were made to you or to your institution)**
**1**	All support for the present manuscript (e.g., funding, provision of study materials, medical writing, article processing charges, etc.) **No time limit for this item.**	☐ **None**	
		NIH Grant No. R01 AG060470	To institution
			Click the tab key to add additional rows.
		**Time frame: past 36 months**	
**2**	Grants or contracts from any entity (if not indicated in item #1 above).	☐ **None**	
		NIH Grant No. R01 AG059329	To institution
		NIH Grant No. R01 AG054442	To institution
**3**	Royalties or licenses	☒ **None**	

		Name all entities with whom you have this relationship or indicate none (add rows as needed)	Specifications/Comments (e.g., if payments were made to you or to your institution)
**4**	Consulting fees	☒ **None**	
**5**	Payment or honoraria for lectures, presentations, speakers bureaus, manuscript writing or educational events	☒ **None**	
**6**	Payment for expert testimony	☒ **None**	
**7**	Support for attending meetings and/or travel	☒ **None**	
**8**	Patents planned, issued or pending	☒ **None**	
**9**	Participation on a Data Safety Monitoring Board or Advisory Board	☐ **None**	
		Member of Health and Research Advisory Council of Alzheimer's Los Angeles	No financial relationship
**10**	Leadership or fiduciary role in other board, society, committee or advocacy group, paid or unpaid	☒ **None**	

		Name all entities with whom you have this relationship or indicate none (add rows as needed)	Specifications/Comments (e.g., if payments were made to you or to your institution)
**11**	Stock or stock options	☒ **None**	
**12**	Receipt of equipment, materials, drugs, medical writing, gifts or other services	☒ **None**	
**13**	Other financial or non-financial interests	☒ **None**	

Please place an "X" next to the following statement to indicate your agreement:

☒ I certify that I have answered every question and have not altered the wording of any of the questions on this form.
